# Alterations in Immunomodulatory Potential of ADSCs Undergoing Osteogenic Differentiation in the Context of Future Therapeutic Applications

**DOI:** 10.3390/cells15070614

**Published:** 2026-03-30

**Authors:** Ilona Szabłowska-Gadomska, Stefan Rudziński, Agnieszka Mroczko, Beata Mrozikiewicz-Rakowska, Dominik Cysewski, Piotr Gasperowicz, Katarzyna Bocian

**Affiliations:** 1 Laboratory for Cell Research and Application, Medical University of Warsaw, 02-097 Warsaw, Poland; stefan.rudzinski@wum.edu.pl (S.R.); agnieszka.mroczko@wum.edu.pl (A.M.); 2 Department of Endocrinology, Centre of Postgraduate Medical Education, Bielanski Hospital, 01-809 Warsaw, Poland; beata.mrozikiewicz-rakowska@cmkp.edu.pl; 3 Clinical Research Centre, Medical University of Bialystok, 15-276 Bialystok, Poland; dominik.cysewski@umb.edu.pl; 4 Department of Medical Genetics, Medical University of Warsaw, 02-106 Warsaw, Poland; piotr.gasperowicz@wum.edu.pl; 5 Department of Immunology, Institute of Experimental Zoology, Faculty of Biology, University of Warsaw, 02-096 Warsaw, Poland; k.bocian@uw.edu.pl

**Keywords:** adipose-derived mesenchymal stem/stromal cells, osteogenic differentiation, immunomodulation, proteomics, cell therapy, regenerative medicine

## Abstract

Background: Adipose-derived mesenchymal stem/stromal cells (ADSCs) are gaining recognition in regenerative medicine for their potential for adipogenic, osteogenic, and chondrogenic differentiation, as well as their immunomodulatory properties. However, ADSC-based therapies focus either on differentiation for tissue replacement or on counteracting unrestrained inflammation to prevent tissue destruction and initiate regeneration. Here, we aim to examine the immunomodulatory potential of osteogenically differentiated ADSCs by analyzing their proteomic profile. Methods: Using LC-MS/MS, we generated the proteomic profiles of differentiated and undifferentiated ADSCs and compared them with the Reactome database. Transcriptomic analysis was also performed and compared with the proteomic profile. Results: Comparison of the proteomic (499 up-regulated; 355 down-regulated) and transcriptomic (212 up-regulated; 232 down-regulated) profiles showed 60.1% concordance—both proteins and transcripts showed the same trend. Significantly upregulated proteins in differentiating ADSCs (−log_10_ *p* > 5 and >10) were grouped into four categories: propensity for osteogenic differentiation; immunomodulation/immune/inflammatory response; cell senescence; and cell cycle regulation. Among those proteins, thirteen were reported to play roles in processes such as immunomodulation, inflammatory signaling, or transplant rejection. Conclusions: We observed that differentiating ADSCs might still exert immunomodulatory effects, which could be used in the treatment of, e.g., bone defects.

## 1. Introduction

In cases of severe trauma, degenerative diseases, congenital disorders, and cancer, especially when bone loss is substantial, the tissue regeneration process may be impaired and require specialized medical procedures, such as bone grafting. Given the limited availability of bone material, new solutions are being sought to overcome or alleviate these limitations of standard therapies [1,2].

Adipose-derived stromal/stem cells (ADSCs) represent a population of multipotent mesenchymal stromal/stem cells (MSCs) found in adipose tissue. They play a significant role in tissue development, homeostasis, repair, and regeneration. Due to their abundance, relative ease of access, ability to proliferate in vitro, and, most importantly, their immunomodulatory potential [3,4] and low immunogenicity [5,6,7], they are a valuable tool in regenerative medicine. Although ADSCs have already been included in clinical trials, as evidenced by the number of completed clinical studies (225 as of 26 February 2026, searched after “adipose-derived stem cells” keyword followed by “Study status: completed” and “Study type: all studies” filters), well-documented evidence and a full understanding of their mechanisms of action are still lacking [8,9,10]. For many years, MSCs have been explored in regenerative medicine due to their potential for multidirectional cell differentiation (including osteogenic) to regenerate or replace damaged cells and tissues [11,12]. Today, with the increasing number of immune-mediated diseases, more attention is being paid to their immunological properties and the pro-regenerative effects of MSC on immune system cells [7,13,14,15]. ADSCs are reported in the literature to have low immunogenicity, which is why they can be administered in an allogeneic setting [4,7,16] and have strong immunomodulatory properties [3,4,7,17]. However, in the case of differentiated ADSCs, such information is scarce. Research on MSC differentiation is inconclusive. Some studies suggest that MSCs’ immunomodulatory abilities may change during differentiation. Experiments on the chondrogenic differentiation of murine MSCs isolated from bone marrow (BMSC) have shown that cell specialization can increase MSC immunogenicity, leading to stimulation of dendritic cells [18,19]. Research on the osteogenic differentiation of MSCs from patients with ankylosing spondylitis (AS) has demonstrated an increased osteogenic potential of these cells compared to those from healthy donors. Further observations led Xie et al. (2017) to hypothesize that osteogenic differentiation of MSCs isolated from patients with AS may induce inflammation, as it was associated with increased secretion of the pro-inflammatory chemokine [20]. Another team demonstrated that during osteogenic differentiation of BMSCs from healthy donors, the range and level of expression of selected factors, including IL-6 and CCL2, also changed [21]. On the other hand, research on the immunogenicity of BMSCs has shown that under in vitro conditions, the cells retain their immune-evasive and immunomodulatory properties after differentiation [4,22]. However, there is little to no evidence on this topic for osteogenically induced ADSCs.

Our research presented here focuses on the hypothesis that ADSCs retain their immunomodulatory properties during the differentiation process. For this study, comparative transcriptomic and proteomic analyses of differentiating and undifferentiated ADSCs, isolated from four donors, were performed. This preliminary study provides results on the anti-inflammatory and pro-inflammatory characteristics and immunomodulatory properties of osteogenically differentiating ADSCs, highlighting their therapeutic potential in the context of bone regeneration.

## 2. Materials and Methods

### 2.1. Cell Isolation, Cell Culture, and Differentiation

Adipose-derived stem/stromal cells (ADSCs) were isolated from lipoaspirates obtained with adherence to the Bioethics Committee approvals no. KB/188/2020 and 87/PB/2020 using an enzymatic method previously described [23]. The cells were subsequently expanded and cryopreserved in liquid nitrogen. After thawing, ADSCs were cultured in standard medium (SM): DMEM (Thermo Fisher Scientific, Waltham, MA, USA; catalog no. 31885049) + 10% FBS (Thermo Fisher Scientific, Waltham, MA, USA; catalog no. 10500064) and 1% antibiotic/antimycotic solution (AAS; Corning Inc., Corning, NY, USA; catalog no. 30-004-CI) or NutriStem^®^ (Biological Industries, Beit-Haemek, Israel; basal medium catalog no. 05-200-1, supplement catalog no. 05-201-1) containing 0.1% AAS, until 70% confluence was reached. Cells at passage 3 were then detached and seeded into T25 culture flasks (125 × 10^3^ cells/flask) and 24-well plates (5 × 10^3^ cells/well) in SM. After 24 h, the medium was replaced with osteogenic differentiation medium (OM): DMEM (Thermo Fisher Scientific, Waltham, MA, USA, catalog no. 31885049) supplemented with 10% FBS (Thermo Fisher Scientific, Waltham, MA, USA; catalog no. 10500064), 50 µg/mL ascorbic acid 2-phosphate (Sigma-Aldrich, Saint Louis, MO, USA; catalog no. A8960), 100 nM dexamethasone (Sigma-Aldrich, Saint Louis, MO, USA; catalog no. D2915), 3 mM NaH_2_PO_4_ (Sigma-Aldrich, Saint Louis, MO, USA; catalog no. S5011) + 1% AAS [24,25]. Cells were incubated under standard culture conditions (21% O_2_, 5% CO_2_) in OM, with the medium changed every 2–3 days. Cells maintained in SM served as experimental controls. After 10 days of incubation in either OM or SM, cells from flasks were detached, washed with PBS (Thermo Fisher Scientific, Waltham, MA, USA) without Ca^2+^/Mg^2+^, pelleted, and stored at −80 °C until further analysis. Cells cultured in 24-well plates were assessed for metabolic activity using the Presto Blue assay (Invitrogen, Thermo Fisher Scientific, Waltham, MA, USA; catalog no. A13262), washed with PBS without Ca^2+^/Mg^2+^, fixed in 4% paraformaldehyde (pH = 7.4), and stained with Alizarin S Staining Quantification Assay (ScienCell Research Laboratories, Inc., Carlsbad, CA, USA; catalog no. 8678) to confirm calcium deposition indicative of osteogenic differentiation. Staining was observed under a Nikon Eclipse TE2000-U microscope (10× magnification) (Nikon, Tokyo, Japan).

### 2.2. Proteomic Analysis

#### 2.2.1. Sample Preparation and Proteomics

For proteomic analysis, ADSCs from four independent donors were analyzed (*n* = 4 biological replicates per condition, OM vs. SM). Cell pellets were lysed in 5% sodium deoxycholate buffer containing TCEP and CAA, supplemented with protease and phosphatase inhibitors, followed by sonication and incubation at 60 °C. Protein concentration was determined by BCA assay, and 25 µg of protein was processed with a modified FASP protocol employing 30 kDa filters. After trypsin/LysC digestion, peptides were purified on Waters HLB plates (Waters Corporation, Milford, MA, USA), eluted with 70% acetonitrile/0.1% TFA, dried, and stored at −80 °C.

#### 2.2.2. LC-MS/MS Analysis

Approximately 1 µg of peptides was separated on a Waters C18 column (0.3 mm × 150 mm) (Waters Corporation, Milford, MA, USA) using a Micro M5 repo LC system (SCIEX, Framingham, MA, USA) at 5 µL/min. MS analysis was performed on a ZenoTOF 7600 (SCIEX, Framingham, MA, USA) equipped with an OptiFlow Turbo V ion source (spray voltage 5 kV, 200 °C, curtain gas 35 psi, gas1 20 psi, gas2 60 psi) (SCIEX, Framingham, MA, USA). Data-dependent acquisition (top 20–100 precursors) with dynamic background subtraction was employed. MS1 accumulation time was 100 ms, and MS/MS scans were acquired at 30 V CE with 5 ms accumulation.

#### 2.2.3. Data Analysis

Raw MS data were processed in MaxQuant v2.6.5.0 against the UniProt human proteome (downloaded 1 June 2024; 83,413 entries) [26]. Protein identifications and LFQ values were obtained using standard parameters. Contaminants, reverse hits, proteins identified only by site or by a single peptide, and inconsistently quantified proteins were removed. Three LC–MS/MS runs were excluded a priori due to persistent technical failure (poor chromatographic performance with low MS signal and markedly reduced identification depth despite technical re-runs), leaving 8 SM and 9 OM samples for downstream LFQ analysis in LFQ-Analyst (log_2_ LFQ; MNAR imputation, downshift 1.8 SD/width 0.3; limma empirical Bayes; Benjamini–Hochberg FDR ≤ 0.05 with |log_2_FC| ≥ 1).

### 2.3. Transcriptomic Analysis

RNA was isolated from cell pellets using the AllPrep DNA/RNA Mini Kit (QIAGEN, Venlo, The Netherlands). RNA concentration was measured with a Qubit Fluorometer (Thermo Fisher Scientific, Waltham, MA, USA), and integrity was assessed by capillary electrophoresis; only samples with an RNA Integrity Number (RIN) ≥ 8 was processed for sequencing.

RNA-seq libraries were prepared using the TruSeq Stranded mRNA kit (Illumina, San Diego, CA, USA) and sequenced on a NovaSeq 6000 (Illumina, San Diego, CA, USA) in paired-end mode (2 × 100 bp), targeting 20–50 million read pairs per sample. Quality control was performed using FastQC v.0.11.9, followed by adapter trimming with BBDUK2 (part of BBTools 36.67). Transcript quantification was carried out using Salmon v.0.12 against the human hg19 reference transcriptome, generating Transcripts per Million values.

### 2.4. Statistical Analysis

#### 2.4.1. ADSC Proliferation

Raw fluorescence intensities were used to calculate the fold-change in ADSC proliferation rate, using undifferentiated ADSCs as a reference. Fold-changes were pooled in the respective groups and compared using the Mann–Whitney test in GraphPad Prism version 9.5.1 for Windows, GraphPad Software (GraphPad Software LLC, Boston, MA, USA).

#### 2.4.2. Proteomic Data Statistical Analysis

Data were analyzed with the LFQ Analyst platform [27]. Proteomic material was obtained from four independent donors and cultured under the indicated conditions (SM vs. OM, with the corresponding media backgrounds). A subset of LC–MS/MS runs was excluded due to technical acquisition failures (poor chromatographic performance/low MS signal and markedly reduced identification depth despite technical re-runs); after these QC-based exclusions, the final dataset comprised 8 SM (undifferentiated) and 9 OM (differentiated) runs used for downstream analysis. Excluding these runs also avoided situations where missingness would exceed ~50% within a sample and make results strongly imputation-driven, which is analytically problematic in MS-based datasets [28].

LFQ intensities were log_2_-transformed, and missing values imputed using the MNAR method (Gaussian distribution, shift 1.8 SD, width 0.3). Differential expression was assessed with limma v.3.58 (R Bioconductor v.3.20), applying a Benjamini–Hochberg FDR ≤ 0.05 and |log_2_ fold-change| ≥ 1. MaxQuant analysis quantified 2.743 proteins, of which 324 were significantly regulated (FDR ≤ 0.05, |log_2_ fold-change| ≥ 1). For broader biological interpretation, a more permissive cutoff (*p* < 0.05, no fold-change threshold) was applied, yielding 854 proteins. Downstream functional analyses included Reactome pathway analysis and STRINGdb network analysis [29,30].

#### 2.4.3. Transcriptomic Data Statistical Analysis

Differential gene expression analysis was performed in R v4.4.0 using DESeq2 v1.44.0 (Bioconductor v3.19). Raw read counts were normalized using DESeq2’s median-of-ratios method, and differential expression was assessed between undifferentiated and differentiating cell populations (*n* = 12 per group—four ADSC donors cultured in three independent cell cultures). Genes with an adjusted *p*-value < 0.05 and |log_2_ fold-change| > 1 were considered significantly differentially expressed. Principal component analysis was conducted on variance-stabilized transformed data to evaluate sample clustering and experimental reproducibility. Statistical analyses and visualizations were generated using the ggplot2 package (v3.5.1).

## 3. Results

### 3.1. ADSC Osteogenic Differentiation

**Figure 1:**
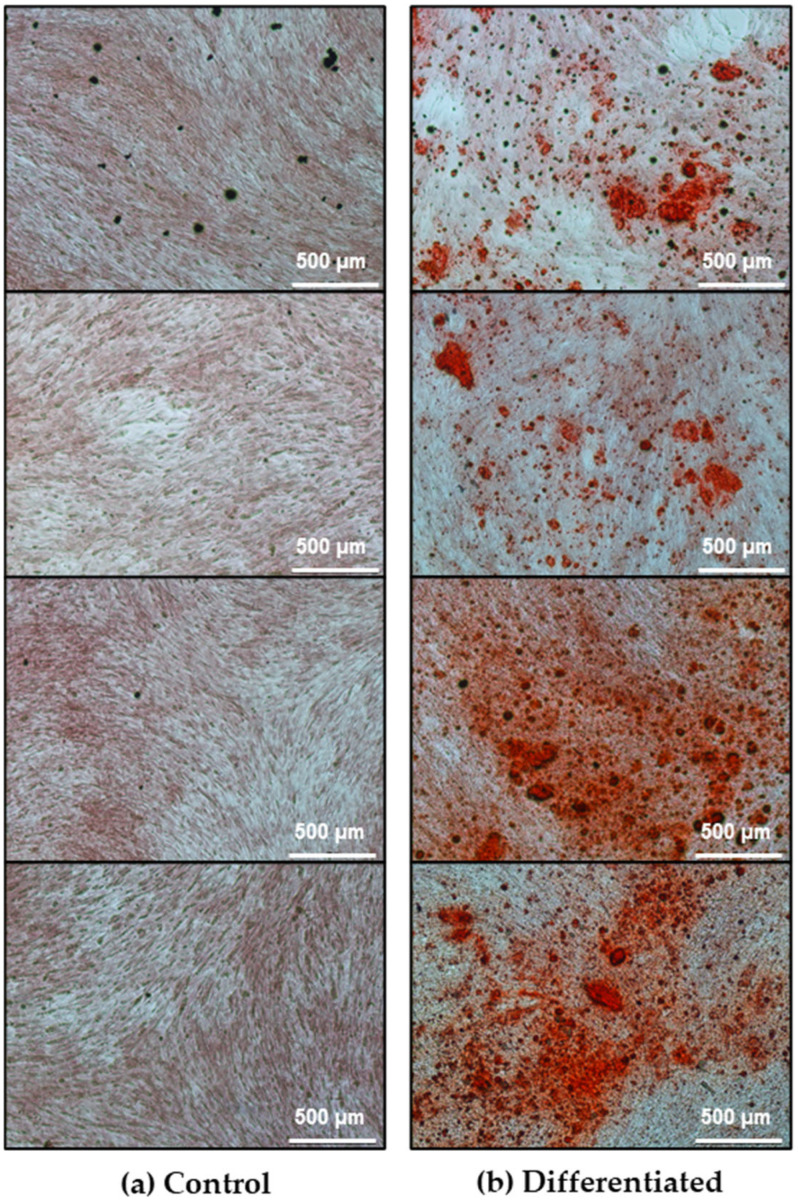
Assessment of the progression of ADSCs’ osteogenic differentiation after 10 days of culture using Alizarin Red staining. (**a**) Control ADSCs cultured in SM; (**b**) differentiating ADSCs cultured in OM. Calcium deposits are visible as red spots.

**Figure 2:**
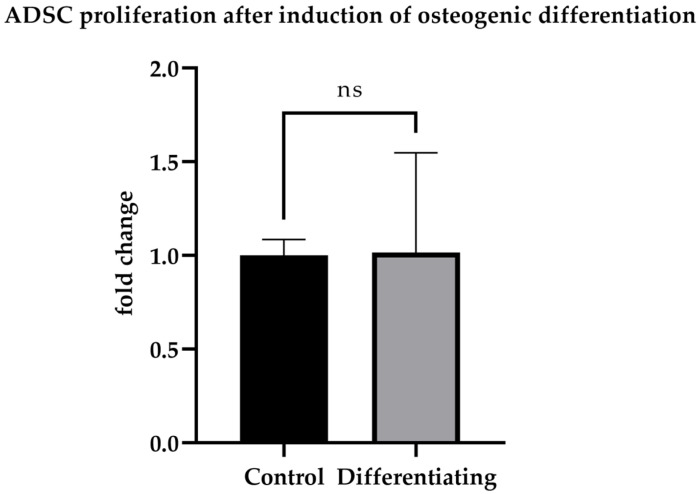
Presto Blue analysis of ADSCs. Results for ADSCs cultured in OM (*n* = 60 per group—four ADSC donors, in 15 technical replicates; mean = 1.016; SEM 0.06877) are presented as the mean relative fold-change calculated from raw fluorescence, with control of ADSCs cultured in SM (*n* = 60 per group—four ADSC donors, in 15 technical replicates; mean = 1.000; SEM = 0.01103) used as the reference. (ns = not significant).

After 10 days of culture, ADSCs were assessed for osteogenic differentiation. ADSCs cultured in osteogenic medium (OM) exhibited calcium deposits, visible as red spots after Alizarin Red staining (Figure 1), whereas undifferentiated ADSCs cultured in standard medium (SM) showed no detectable calcium deposits. The Presto Blue assay revealed no significant differences in proliferation rates between differentiating and undifferentiated ADSCs; however, cells in OM displayed greater variability (Figure 2).

### 3.2. Transcriptomic Analysis of Differentiating ADSCs

**Figure 3:**
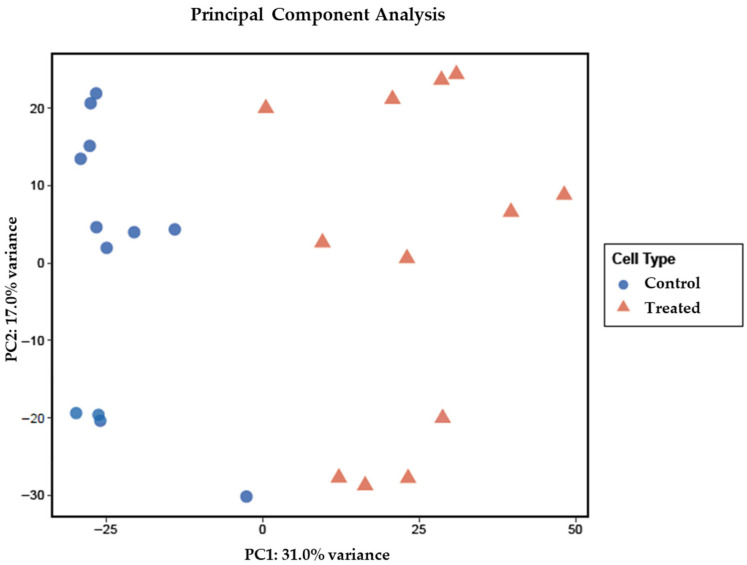
Principal Component Analysis of gene expression comparing undifferentiated ADSCs (control) and differentiating ADSCs (treated).

The transcriptomic analysis revealed substantial molecular reprogramming during osteogenic differentiation, with over 16,000 genes differentially expressed between differentiating and undifferentiated ADSCs (Table S1). Principal component analysis (PCA) demonstrated clear separation between these two experimental groups along PC1 (31.0% variance) and PC2 (17.0% variance), confirming distinct transcriptional profiles associated with the differentiation process. Notably, while the control and treated samples formed distinguishable clusters, considerable inter-patient variability was observed within each group (Figure 3), suggesting that individual donor characteristics contribute significantly to the overall transcriptomic landscape.

### 3.3. Transcriptome–Proteome Concordance

**Figure 4:**
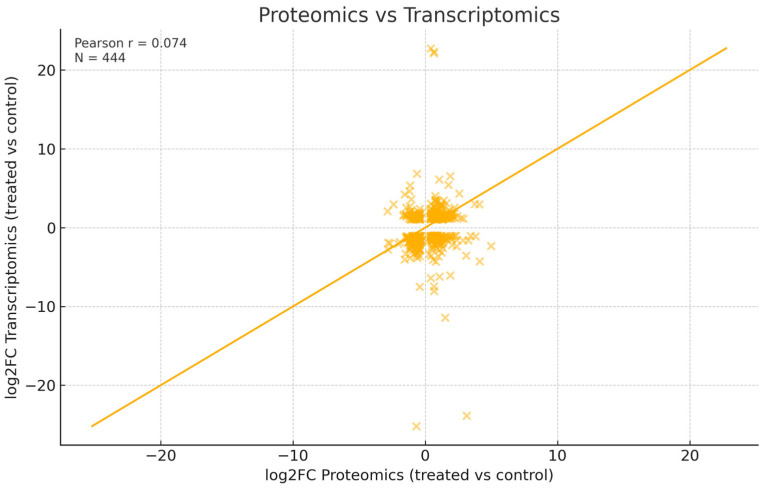
Scatter plot showing the correlation between proteomic and transcriptomic fold-changes in osteogenically differentiating ADSCs (treated vs. control) based on 444 paired features. The diagonal line represents y = x. Pearson correlation was very weak (r = 0.074), indicating low concordance between expression layers and suggesting post-transcriptional regulation during differentiation.

Across the proteomics-significant dataset (N = 854), upregulated proteins predominated (N = 499; 58.4%), consistent with the activation of ECM deposition/remodeling during osteogenic differentiation (Table 1). The scatter plot analysis of proteomic and transcriptomic fold-changes revealed a very weak linear correlation (Pearson r = 0.074; Figure 4), consistent with the typically weak mRNA–protein correlations reported in differentiation studies. Within the corresponding transcriptomic subset (N = 444 pairs), the distribution was more balanced (47.7% upregulated vs. 52.3% downregulated). The trend analysis in the proteomics–RNA pairs showed moderate concordance. Among 444 matched protein–RNA pairs, 60.1% were concordant in direction (32.4% both up, 27.7% both down), while 39.9% were discordant (Table 2). This pattern is typical of differentiation, where post-transcriptional regulation, protein secretion, and matrix accumulation kinetics dampen the one-to-one correspondence between mRNA and protein levels. From a biological perspective, the dataset of concordant identifications between transcriptomics and proteomics supports two key axes of our study: the osteogenic axis related to ECM/mineralization—COL8A1, COL6A2, MGP, COL3A1, CCN2/CTGF, and the immunomodulatory axis—B2M, CTSB, CTSD, ADGRE5/CD97, and STAT2.

### 3.4. Proteomic Analysis of Differentiating ADSCs

**Figure 5:**
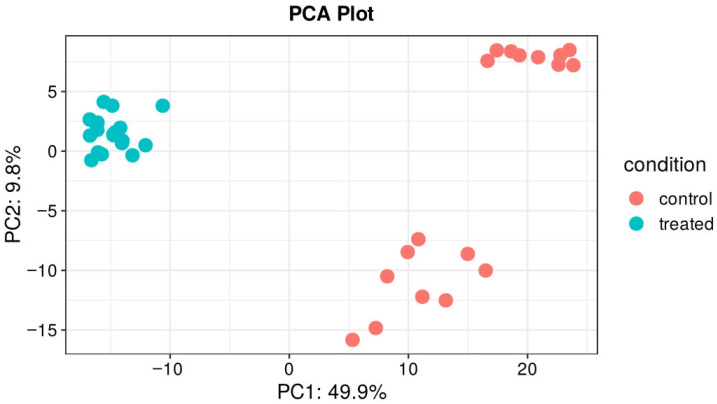
Principal Component Analysis of protein expression comparing undifferentiated ADSCs (control) and differentiating ADSCs (treated). The analysis shows that the differentiation process increased the homogeneity of the proteomic profile and led to substantial changes relative to undifferentiated ADSCs.

**Figure 6:**
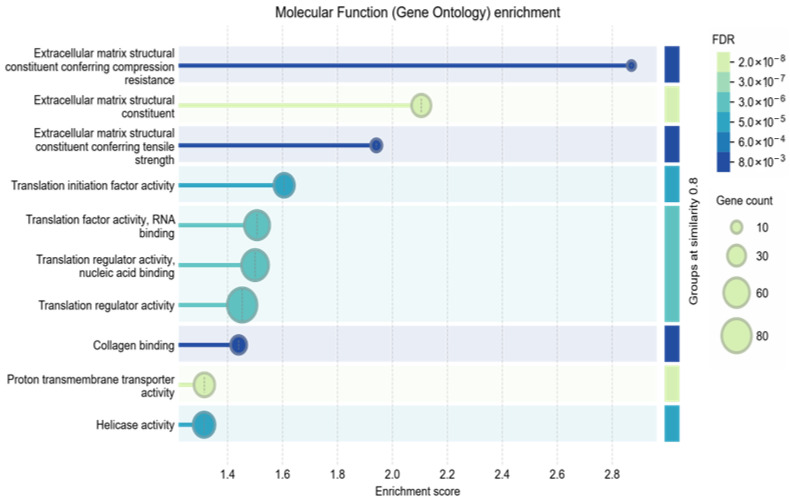
Analysis of proteins with upregulated expression in differentiating ADSCs, indicating enrichment in processes related to extracellular matrix organization and enhanced transcriptomic activity.

**Figure 7:**
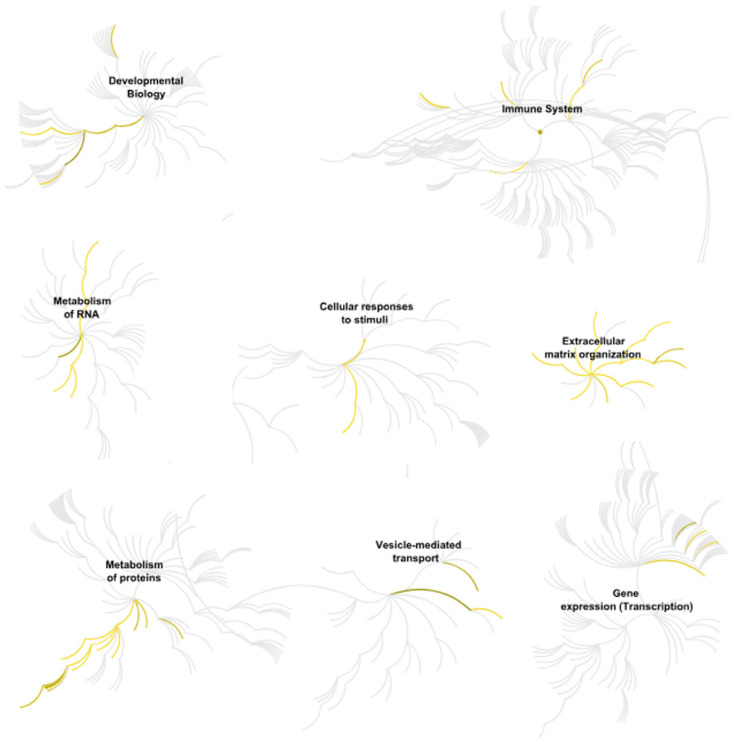
Selected pathways enriched in osteogenically differentiating ADSCs showing the strongest significance and enrichment according to the Reactome database.

Given the exploratory, hypothesis-generating scope and the limited donor cohort, we emphasize biological coherence at the pathway/process level rather than isolated single-protein claims, while restricting any protein-level statements to the BH-FDR-controlled results and treating relaxed-threshold protein lists as descriptive context only. The comparison of proteomic profiles of undifferentiated ADSCs (SM) and cells cultured in osteogenic medium (OM) showed clear differences in protein abundance after ten days of culture. Principal component analysis (PCA) of significantly regulated proteins separated from the OM and SM groups, with higher within-group similarity observed for the OM samples (Figure 5). Functional enrichment of upregulated proteins identified molecular functions related to extracellular matrix (ECM) assembly and collagen binding (Figure 6), in line with the presence of calcium deposits detected by Alizarin Red staining (Figure 1). Reactome pathway analysis indicated enrichment of the terms “extracellular matrix organization,” “developmental biology,” and “cellular response to stimuli” in OM-treated ADSCs (Figure 7).

**Figure 8:**
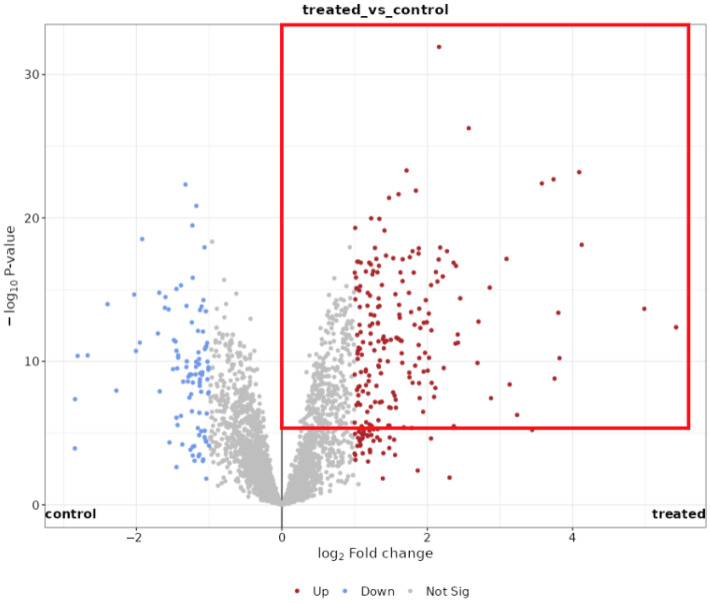
Volcano plot of differentially expressed proteins in osteogenically differentiating ADSCs (treated vs. control). Proteins meeting the significance criteria Benjamini–Hochberg FDR ≤ 0.05 and |log_2_ fold-change| ≥ 1 are shown as downregulated (blue) and upregulated (red). Upregulated proteins with −log_10_ *p* > 5 are additionally highlighted in the red box as a visual annotation tier (see Section 2.4.2 for the inferential criteria).

Pathway annotation grouped the significantly regulated proteins into four main biological categories: Propensity for Osteogenic Differentiation, Immunomodulation/Immune/Inflammatory Response, Cell Senescence, and Cell Cycle Regulation (Table 3). This study is exploratory and hypothesis-generating; therefore, statistical inference is restricted to FDR-controlled results, while relaxed thresholds are used only for descriptive pathway context and are interpreted cautiously. In Figure 8, the −log_10_ *p* > 5 and >10 lines are shown only as visual-based annotation tiers chosen heuristically from the volcano-plot. When more stringent significance thresholds were applied (−log_10_ *p* > 5 and >10), these pathway categories remained enriched (Figure 8).

From the list of proteins that were significantly upregulated in differentiated ADSCs we curated a list of 13 for which the literature reports roles in immunomodulation, inflammatory signaling, transplant rejection, or graft-versus-host disease (Table 4).

Overall, the proteomic analysis of osteogenically induced ADSCs revealed a profile characterized by increased abundance of proteins associated with extracellular matrix organization, collagen binding, and immune-related processes.

## 4. Discussion

In our study, we observed early signs of osteogenic differentiation after 10 days, confirmed by calcium deposits and proteomic changes. Within the proteins related to osteogenic pathways, small leucine-rich proteoglycans and matricellular proteins were prominent. Biglycan (BGN) supports BMP-dependent osteoblast differentiation and matrix mineralization [32]. Decorin (DCN) promotes osteogenic differentiation via ERK1/2 signaling [33]. Lumican (LUM) enhances osteoblastogenesis and suppresses bone resorption [34,64]. Matrix Gla protein (MGP) regulates osteoblast proliferation, differentiation, and mineralization through Wnt/β-catenin signaling [35]. Together with CCN2/CTGF, these proteins anchor the ECM-driven mineralization axis of ADSC osteogenesis. Although some of the ECM-related changes detected after 10 days could partially reflect the adaptation of ADSCs to in vitro differentiation induction, several studies indicate that even a 7-day time frame is sufficient for stimulus-dependent ECM remodeling, including early increases in type I collagen, osteopontin, or decorin expression [65,66,67]. Therefore, the alterations observed in our study are more likely to represent an early, cue-specific response rather than passive adaptation.

Longer osteogenic induction periods (14–21 days) are not aligned with regulatory expectations, as extended culture reduces MSC functionality [68]. The 10-day window used here aligns with time frames considered safe and practical for translational MSC workflows and allows the assessment of how short-term differentiating treatment influences functionally relevant properties.

Alizarin Red staining serves only as an auxiliary readout of early ECM–mineral interactions. Our aim was not full osteogenesis but rather to determine whether osteogenic cues alter ADSC immunomodulation. The ECM-related responses observed at day 10 should therefore be interpreted as early, non-terminal events [66,67,69,70,71].

OM-treated ADSCs displayed an immunomodulatory signature, more pronounced in the proteome than in the transcriptome (Figure 4). ADSCs showed increased homogeneity in their proteomic profile after the induction of the osteogenic differentiation, as shown in PCA analysis (Figure 5). Thus, we decided to increase the range of proteomic analysis to log_2_ fold-change over 1 rather than 2 (Figure 8, red box), which allowed us to widen the view of changes in the immunomodulatory potential of ADSCs. Proteins involved in antigen presentation and adhesion were increased, including β2-microglobulin (B2M), HLA-A, HLA-C, and ICAM-1. MHC class I complexes are central to CD8^+^ T-cell surveillance [72], while ICAM-1 mediates leukocyte adhesion and immune synapse formation [73]. ADGRE5/CD97 supports leukocyte trafficking and inflammatory regulation [74]. Lysosomal cathepsins (CTSB, CTSD) were also upregulated, consistent with matrix remodeling and immune activation [61,75]. Additional immune-linked factors include STAT3, STAT2, LTF, LTA4H, CAT, SOD2, and LGALS3, several of which are well-documented in immune regulation and regenerative processes (Table 2).

Bone regeneration depends on coordinated interactions between immune and skeletal cells, as emphasized in osteoimmunology. This field investigates how immune mechanisms influence the physiology and pathology of bone. Research in osteoimmunology shows that balanced communication among pre-osteoblasts, osteoblasts, osteocytes, osteoclasts, and immune cells ensures the proper progression of all phases of bone repair. Conversely, dysregulation of immune–skeletal crosstalk contributes to bone disorders, including non-union fractures, autoimmune-driven bone loss, and regeneration deficits associated with aging, chronic disease, or treatments such as corticosteroids [76,77,78,79]. Interestingly, one of the most commonly used factors for osteogenic differentiation induction is dexamethasone, a potent glucocorticoid also used in our OM [80,81]. While the fact that we did not include a control variant for dexamethasone should be noted as a limitation of our work, the concentration used in OM, namely 100 nM, is magnitudes lower than those reported to exert any immune-related pharmacological effects in ADSCs [82]. The proteomic profile of the ADSCs differentiated in our study indicates the generation of osteogenic progenitors/osteoblast precursors corresponding to pre-osteoblasts. These cells exhibit the potential to actively drive the bone formation phase of the remodeling process, while simultaneously retaining the capacity to modulate immune responses and dynamically communicate with cells of the immune system. Proteomics clearly separated the groups, while transcriptomic differences were less prominent, consistent with the low RNA–protein correlation reported in many studies [83,84,85]. This pattern is considered typical and is attributed to post-transcriptional regulation, differences in protein turnover, and temporal delays between transcriptional and translational responses [83,86].

It is well established that only a fraction of mRNA changes is reflected at the protein level. In many studies, about half or slightly more genes show concordant regulation between transcriptomics and proteomics due to numerous regulatory events that take place between mRNA synthesis and translation into proteins [84,85,87,88,89]. For example, Koussounadis et al. reported that typically only 0–50% of protein-level differences are accompanied by consistent changes at the mRNA level [90]. Similarly, analyses of genetic effects on expression showed that ~64% of mRNA–protein changes occur in the same direction, only slightly above the random expectation of 50% [91]. Therefore, the observation of ~60% concordance between RNA and protein directions is consistent with the consensus in the literature, reflecting a typical degree of overlap between transcriptome and proteome regulation. This moderate level of agreement reflects the multiple levels of regulation beyond transcription that determine protein abundance [83]. We note that discordance can arise from multiple non-exclusive sources (kinetics, donor variability, transient responses), as well, which cannot be separated in our dataset.

MSCs, including ADSCs, are widely investigated in bone regeneration due to their osteogenic potential and proliferative capacity [25,92]. Regardless of whether auxiliary systems, such as various scaffolds, are used, the clinical application of ADSCs for bone regeneration follows two main paths—the use of undifferentiated ADSCs or their pre-differentiation in vitro before ADSC transplantation into the bone tissue [93,94,95]. The use of undifferentiated ADSCs is easier to implement clinically, as it requires less manipulation of cells and therefore follows less restrictive quality assessment processes. However, undifferentiated ADSCs are usually paired with various scaffolds, often loaded with bone-regeneration-promoting growth factors such as BMP-2, BMP-4, or TGF-beta, which may pose risks to patients [94,96]. Moreover, transplanted undifferentiated ADSCs show tendencies toward increased expression of stemness-related genes, resulting in lower differentiation potential than that of cells cultured in vitro [92,97]. Thus, they may be less predictable than cells already primed towards differentiation. On the other hand, the use of pre-differentiated in vitro ADSCs enhances bone regeneration without the need for scaffolds or additional growth factors during application. Promising results were demonstrated in in vivo models where pre-differentiated ADSCs were used with or without scaffolds [98,99,100]. Interestingly, Dufrane et al. (2015) successfully isolated autologous ADSCs from 6 underage patients with bone nonunion, expanded them in vitro, and osteogenically differentiated them to the 4th passage [101]. Differentiated ADSCs were then mixed with demineralized bone matrix to create 3 × 3 cm^2^ autografts, which were subsequently transplanted into the fracture site. No treatment-related adverse events were reported, and 3 out of 6 patients showed confirmed bone consolidation, demonstrating the feasibility of this approach. Most differentiation protocols suggest that ADSCs have to be cultured for 14–21 days to achieve osteogenic differentiation [25]. However, prolonged cell culture introduces the risk associated with the loss of ADSC stability compared with less manipulated cells. In our study, ADSCs showed the first signs of induced differentiation after only 10 days, and Zhu et al. (2021) reported positive osteogenic differentiation of ADSCs and DPSCs (dental pulp stem cells) after just 7 days [102,103].

The use of in vitro pre-differentiated ADSCs as a pro-regenerative agent in bone regenerative therapies raises questions regarding the safety and stability of cultured cells. Our studies showed that prolonged (10-day) cell culture in OM did not show, in the proteomic analysis, induction of HLA-B expression and simultaneously affected other MHC I molecules, namely HLA-A and HLA-C. The estimated fold-change was 1.7 and 2.07 for HLA-A and HLA-C, respectively.

The proteomic method used in our study is not suitable for exploring the crosstalk of the HLA-A: HLA-C ratio. However, changes in MHC I expression, together with increased TGFBI, suggest a shift toward a more anti-inflammatory signature [104,105]. The question remains whether this protolerogenic immunophenotype is sufficient to help mitigate the pro-inflammatory conditions typically associated with bone remodeling and regenerative processes. Further mechanistic studies on a bigger donor base with well-documented data are needed to confirm those observations.

In their study, Dufrane et al. (2015) also reported that ADSCs showed fewer karyotypic changes after differentiation induction than before, suggesting that the differentiation process renders ADSCs safer [101]. The partially activated MHC I complex may be related to ECM remodeling processes, as seen in upregulation of TGFBI, which usually accompanies osteogenic differentiation, including collagen deposition. Some studies suggest that B2M, a component of the MHC I complex, is associated with collagen production, M1 macrophage modulation, and regeneration processes, potentially linked to TGF-beta signaling activated during osteogenic differentiation [103,106,107,108].

The bone formation phase fundamentally relies on the polarization of macrophages toward the M2 phenotype and the inhibition of osteoclast activity, a process associated with the presence of factors such as TGF-β. In osteoporosis therapy, the most desirable cells are those that intrinsically exhibit high proliferative potential and the capacity to differentiate into osteoblasts, while simultaneously suppressing osteoclast function and formation, and thus the process of bone resorption [109,110]. It also appears that the cells obtained in our study could potentially be used to support pharmacological and physiotherapeutic approaches in ankylosing spondylitis. ADSCs, as allogeneic cells with no increase in HLA-B expression, including the pathological variant HLA-B27, may support bone remodeling and reduce inflammation [111]. The overall proteomic profile of differentiating ADSCs suggests their usefulness in similar therapies, which should be further investigated in future functional studies.

Moreover, in addition to presenting antigens to T lymphocytes, HLA-C receptors regulate the cytotoxic activity of NK cells to promote maternal immune tolerance to fetal antigens [112]. Furthermore, the team led by Prof. M. Siemionov demonstrated a modern personalized therapeutic approach using chimeric myoblasts comprising a donor/patient mix of MHC I and II molecules. The study demonstrated safety and tolerability in all three patients, with no adverse reactions and no elevation of anti-HLA antibodies [113].

We hypothesize that this may be one of the mechanisms by which ADSCs could mitigate potential immunogenicity caused by increased MHC I expression during differentiation. Thus, the use of HLA-C-expressing ADSCs may represent an interesting direction to explore augmentation therapy during transplantations, e.g., bone transplantation, to reduce or potentially eliminate the threat of uncontrolled inflammation. Whether the osteogenically induced ADSCs with clear MHC class I expression are able to mitigate immunogenicity through the same mechanisms remains unexplored. Our study showed that differentiating ADSCs shows a proteomic profile with such potential. However, future studies are required to confirm this hypothesis.

Despite this, the partial activation of the MHC I complex may raise concerns regarding the safety of pre-differentiated ADSCs in bone regeneration therapies. Thus, some researchers are leaning towards exploring “cell-free” approaches, such as extracellular vesicles (EVs) or the ADSC secretome [14,94,114]. In our study, increased CD63 and CD81 protein levels suggest enhanced EV biogenesis [115,116], but functional EV analysis requires dedicated experiments. Moreover, osteogenic differentiation induced expression of STAT2 and STAT3 proteins related to ADSCs’ stemness and their immunomodulatory properties [117,118,119,120,121]. Activation of both metabolic pathways—immunomodulatory signaling and EVs biogenesis—may suggest that osteogenic differentiation does not reduce the immunomodulatory potency of ADSCs but rather stabilizes it by reinforcing crosstalk mechanisms in the remodeling bone environment. However, the cellular extracts are not the right material for EV studies. Future studies involving the changes in the secretome of differentiating ADSCs, with emphasis on EVs production and function, should be performed.

Previous studies confirm strong immunomodulatory properties of ADSCs [122,123], which our proteomic results further support. Most studies use SVF cells or ADSCs for a graft enhancement approach, where bone grafts are accompanied by isolated cells, while others use isolated ADSCs seeded on bone-mimetic biomaterials [124,125]. The growing popularity of ADSCs in regenerative medicine is mainly due to their higher immunomodulatory potential than that of, e.g., BMSCs, as they produce more immunosuppressive cytokines such as IL-6 or TGF-beta [17,126]. In our study, we showed that even cellular ex-tracts of ADSCs that underwent osteogenic differentiation retained immunomodulatory properties.

A recently published study by Zheng et al. (2025) showed in a murine model that ADSCs seeded on fish collagen scaffold induced vascularization and repair at the site of femoral head necrosis [127]. While our data do not directly support similar events, we observed enriched pathways related to extracellular matrix organization and immunomodulation, supported by overexpressed proteins such as STAT3–M2 macrophage polarization, LTF–immunomodulation, and CTSD–extracellular matrix remodeling, repair, and regeneration [47,57,62]. Immunomodulatory events and extracellular matrix reorganization are crucial in bone regeneration and revascularization processes [60,125].

It therefore appears that the ADSCs obtained in our study may support therapies for diseases characterized by autoimmune-driven bone loss, such as rheumatoid arthritis, psoriatic arthritis, systemic lupus erythematosus, or bone loss resulting from osteonecrosis [128,129]. Pre-differentiated ADSCs could serve not only as a pool of cells capable of immediately replenishing the osteoblast population, but also as cells that suppress inflammation induced by hyperactive T lymphocytes while simultaneously supporting angiogenesis within necrotic bone. In rheumatoid arthritis, it is crucial to promote regulatory T cells and inhibit the activation of Th17 lymphocytes and the production of pro-inflammatory cytokines, including IL-17, which recruits macrophages and neutrophils and further enhances osteoclast activity and bone resorption. The application of the cell population identified in our study could help rebalance the phases of bone resorption and formation, while also supporting the restoration of immunological self-tolerance.

Moreover, we observed upregulation of STAT3, ICAM1, LTA4H, and LTF proteins in OM-stimulated ADSCs. Those proteins have been previously reported by others to potentially contribute to the immunomodulatory properties of ADSCs by stimulating the M1/M2 macrophage polarization, controlling neutrophil recruitment, and regulating T-lymphocyte maturation [13,47,49,50,51,52,57,61]. Moreover, upregulated STAT3 expression may indicate the ADSCs’ readiness to participate in bone regeneration processes, particularly those associated with inflammatory responses. STAT3 serves as a signal transducer for several pro-inflammatory and anti-inflammatory cytokines, such as IL-6 or IL-10, respectively [130]. STAT3 phosphorylation is a key event in activating signaling pathways related to osteogenic and osteoclastic processes, macrophage polarization, and angiogenesis [131]. The engagement of STAT3 could suggest that ADSCs cultured in OM activated their pro-regenerative properties, which may be valuable for future cell-based bone therapies. Interestingly, our findings show increased levels of LTA4H, an enzyme typically associated with pro-inflammatory activity, as its intercellular form catalyzes the production of leukotriene B4, a strong neutrophil activator [53]. However, when secreted into the extracellular matrix (ECM), LTA4H cleaves the proline-glycine-proline motif of the bioactive collagen fragment that normally serves as the neutrophil activator, thereby reducing inflammation and controlling pathological ECM remodeling [54,55]. The duality of LTA4H illustrates the potential dynamic nature of ADSC immunomodulation, even during osteogenic differentiation, which should be further investigated.

Interestingly, Li et al. (2024) showed in a murine model that human ADSCs, after inflammatory stimulation, displayed increased expression of SOD, resulting in enhanced immunomodulatory effects on macrophages and T lymphocytes, although at the expense of their adipogenic differentiation capacity [132]. On the other hand, our results show that the SOD protein was upregulated in osteogenically differentiating ADSCs. Increased SOD expression may reflect enhanced antioxidative and immunoregulatory activity during osteogenic induction. Kiernan et al. (2020) demonstrated in vitro that chondrogenically differentiated BMSCs retained the ability to modulate T-lymphocyte populations through IL-6 secretion [133]. The authors hypothesized that this may be one of the mechanisms enabling differentiated MSCs to maintain immune evasiveness. Although we did not perform functional assays, the proteomic profile observed in our study suggests that, during osteogenic differentiation, ADSCs may retain their immunomodulatory properties.

Overall, early-differentiating allogenic ADSCs remain promising for bone regeneration therapies, but further functional studies are necessary. Our study creates a groundwork for further exploration. In particular, the potential adoption of differentiating ADSCs as a standardized therapy requires further investigation, as the immunomodulation is dynamic and highly dependent on the local environment into which the cells are transplanted.

## Figures and Tables

**Table 1:** Differentially regulated proteins and transcripts in osteogenically differentiating ADSCs.

	Total N	Up-Regulated (N, %)	Down-Regulated (N, %)
Proteomics (protein groups)	854	499 (58.4%)	355 (41.6%)
Transcriptomics (transcripts)	444	212 (47.7%)	232 (52.3%)

**Table 2:** Concordance of proteomic and transcriptomic changes in osteogenically differentiating ADSCs.

Category	N	% Paired with RNA
Prot ↑/RNA ↑	144	32.4%
Prot ↓/RNA ↓	123	27.7%
Prot ↑/RNA ↓	109	24.5%
Prot ↓/RNA ↑	68	15.3%
TOTAL Concordant	267	60.1%
TOTAL Discordant	177	39.9%

↑—an indicator of upregulated expression trend in osteogenically induced ADSCs. ↓—an indicator of downregulated expression trend in osteogenically induced ADSCs.

**Table 3:** Processes annotated in the Reactome database with significantly identified proteins (−log_10_ *p* > 5 and >10) and their potential biological role.

Process Name	Protein Name (Gene)	Protein Biological Role	Log_2_ Fold-Change	Ref.
Propensity for Osteogenic Differentiation	CCN2 (Connective Tissue Growth Factor)	CCN2 (CTGF) promotes osteogenic differentiation, enhancing bone matrix proteins, alkaline phosphatase activity, and mineralized nodule formation.	1.0	[31]
	BGN (Biglycan)	Biglycan regulates bone formation, modulates osteoblast differentiation, and matrix mineralization via BMP signaling.	1.05	[32]
	DCN (Decorin)	Decorin enhances osteogenic differentiation via activation of ERK1/2 signaling, facilitating osteoblast maturation.	3.09	[33]
	LUM (Lumican)	Lumican stimulates osteoblast differentiation and suppresses bone resorption, enhancing bone formation.	1.2	[34]
	MGP (Matrix Gla Protein)	MGP positively influences bone formation by promoting osteoblastic cell proliferation, differentiation, and mineralization via the Wnt/β-catenin pathway and Runx2.	1.76	[35]
Immunomodulation/Immune/Inflammatory Response	LTA4H (Leukotriene A4 hydrolase)	LTA4H catalyzes leukotriene B4 synthesis, amplifying inflammation and recruiting neutrophils and leukocytes.	0.468	[36]
	LTF (Lactoferrin)	Lactoferrin is an immunomodulator that limits infections and inflammation by controlling cytokine production and ROS.	1.95	[37]
	CD68 (Macrosialin)	CD68, expressed by macrophages, is a marker of activated phagocytes, indicating chronic inflammation and immune activation.	1.16	[38]
	ADGRE5 (CD97)	CD97 facilitates leukocyte adhesion and migration, supporting granulocyte homeostasis and antibacterial immune responses.	0.868	[39]
	MHC I: HLA-A HLA-C	Presentation of endogenous proteins to Tc lymphocytes, regulation of cytotoxic functions of NK cells. mesenchymal stem cells transfer mitochondria to allogeneic Tregs in an HLA-dependent manner, improving their immunosuppressive activity.	0.775 1.05	[40,41,42,43]
Cell senescence	GLB1 (Galactosidase-β 1, lysosomal)	GLB1 encodes lysosomal β-galactosidase, a marker of senescent cells correlated with increased p16INK4a and reduced proliferation.	0.841	[44]
Cell Cycle Regulation	TBX3 (T-box 3 transcription factor)	TBX3 represses p21WAF1, preventing cell cycle arrest and supporting proliferation, bypassing senescence checkpoints.	1.89	[45]

**Table 4:** Proteins showing strong upregulation in the extended proteomics analysis (−log_10_ *p* > 10) and supported by literature evidence for involvement in immunological processes relevant to regenerative medicine. For each protein, the direction and log_2_ fold-change are shown together with the corresponding transcript level (log_2_ fold-change) and trend, where available. NS indicates that the transcript was quantified but did not reach statistical significance in the RNA-seq group comparison, whereas ND indicates that the transcript was not detected.

Protein	Trends	Log_2_ Fold-Change	ADSC Immunomodulatory/Therapeutic Potential	Reference
STAT3	Prot up	0.97	Immunomodulatory	[46]
	Transcript down	−1.01	Anti-inflammatory	[47]
			GvHD mitigation	[48]
HLA-A	Prot up	0.78	GvHD mitigation	[42]
	Transcript up	0.93, NS		
HLA-C	Prot up	1.05	GvHD mitigation	[41,42,43]
	Transcript ND	ND		
ICAM1	Prot up	1.46	Immunomodulation	[13,49,50,51]
	Transcript NS	0.49, NS	Transplant rejection	[51]
LTA4H	Prot up	0.47	Proinflammatory	[52,53]
	Transcript down	−1.10	Anti-inflammatory	[54,55]
LTF	Prot up	1.95	Immunomodulation	[56]
	Transcript NS	0.99, NS	Anti-inflammatory	[57]
CAT	Prot up	0.68	Anti-inflammatory	[58]
	Transcript NS	−1.21, NS		
SOD2	Prot up	2.86	Anti-inflammatory	[59]
	Transcript down	−1.61		
CTSB	Prot up	1.33	Tissue remodeling	[60]
	Transcript up	1.39		
CTSD	Prot up	1.04	Tissue remodeling	[61]
	Transcript up	2.37		
PTPN11	Prot up	0.56	Immunomodulatory	[62]
	Transcript down	−1.04		
LGALS3	Prot up	1.22	Immunomodulatory	[63]
	Transcript up	1.19		
CD68	Prot up	1.16	Immunomodulatory	[38]
	Transcript up	2.54		

## Data Availability

The proteomic data are deposited in the PRIDE repository under accession number PXD073297, Username: reviewer_pxd073297@ebi.ac.uk, Password: wrKxWmbdQDL2. The transcriptomic data are presented in Table S1 of the Supplementary Materials.

## Supplementary Materials

The following supporting information can be downloaded at: https://www.mdpi.com/article/10.3390/cells15070614/s1, Table S1: RNA-seq differentital expression of osteogenically induced ADSCs vs control ADSCs.

## Abbreviations

AAS	Antibiotic-Antimycotic Solution
ADREG5/CD97	Adhesion G Protein-Coupled Receptor E5
ADSC	Adipose-Derived Stem/Stromal Cell
AS	Ankylosing Spondylitis
B2M	Beta-2-Microglobulin
BCA	Bicinchoninic Acid
BGN	Biglycan
BMP-2	Bone Morphogenetic Protein 2
BMP-4	Bone Morphogenetic Protein 4
BMSC	Bone Marrow Mesenchymal Stem/Stromal Cell
CAA	Chloroacetic Acid
CAT	Catalase
CCL2	C-C Motif Chemokine Ligand 2
CCN2/CTGF	Cellular Communication Network Factor 2
CD63	CD63 Molecule
CD68	CD68 Molecule
CD81	CD81 Molecule
COL3A1	Collagen Type III Alpha 1 Chain
COL6A2	Collagen Type VI Alpha 2 Chain
COL8A1	Collagen Type VIII Alpha 1 Chain
CTSB	Cathepsin B
CTSD	Cathepsin D
DCN	Decorin
DMEM	Dulbecco’s Modified Eagle Medium
DPSC	Dental Pulp Stem/Stromal Cells
ECM	Extracellular Matrix
EV	Extracellular Vesicles
FASP	Filter Aided Sample Preparation
FBS	Fetal Bovine Serum
FDR	False Discovery Rate
GLB1	Galactosidase Beta 1
GvHD	Graft versus Host Disease
HLA-A	Major Histocompatibility Complex, Class I, A
HLA-B	Major Histocompatibility Complex, Class I, B
HLA-C	Major Histocompatibility Complex, Class I, C
ICAM1	Intercellular Adhesion Molecule 1
IL-10	Interleukine 10
IL-6	Interleukine 6
LC-MS/MS	Liquid Chromatography-Mass Spectrometry
LFQ	Label-Free Quantification
LGALS3	Galectin 3
LTA4H	Leukotriene A4 Hydrolase
LTF	Lactotransferrin
LUM	Lumican
MGP	Matrix Gla Protein
MHC I	Major Histocompatibility Complex Class I
mRNA	Messenger Ribonucleic Acid
MSC	Mesenchymal Stem/Stromal Cell
OM	Osteogenic Medium
p16INK4a	Cyclin Dependent Kinase Inhibitor 2A; CDKN2A
p21WAF1	Cyclin Dependent Kinase Inhibitor 1A; CDKN1A
PBS	Phosphate-Buffered Saline
PCA	Principal Component Analysis
PTPN11	Protein Tyrosine Phosphatase Non-Receptor Type 11
RIN	RNA Integrity Number
RNA	Ribonucleic Acid
SM	Standard Medium
SOD	Superoxide Dismutase
SOD2	Superoxide Dismutase 2
STAT2	Signal Transducer And Activator Of Transcription 2
STAT3	Signal Transducer And Activator Of Transcription 3
SVF	Stromal-Vascular Fraction
TBX3	T-Box Transcription Factor 3
TCEP	Tris(2-carboxyethyl)phosphine
TFA	Trifluoroacetic Acid
TGF-beta	Transforming Growth Factor Beta
TGFBI	Transforming Growth Factor Beta Induced
